# Attention-Induced Deactivations in Very Low Frequency EEG Oscillations: Differential Localisation According to ADHD Symptom Status

**DOI:** 10.1371/journal.pone.0017325

**Published:** 2011-03-08

**Authors:** Samantha J. Broyd, Suzannah K. Helps, Edmund J. S. Sonuga-Barke

**Affiliations:** 1 Developmental Brain-Behaviour Laboratory, School of Psychology, Institute for Disorders of Impulse and Attention, University of Southampton, Southampton, United Kingdom; 2 Department of Experimental Clinical and Health Psychology, Ghent University, Ghent, Belgium; Cuban Neuroscience Center, Cuba

## Abstract

**Background:**

The default-mode network (DMN) is characterised by coherent very low frequency (VLF) brain oscillations. The cognitive significance of this VLF profile remains unclear, partly because of the temporally constrained nature of the blood oxygen-level dependent (BOLD) signal. Previously we have identified a VLF EEG network of scalp locations that shares many features of the DMN. Here we explore the intracranial sources of VLF EEG and examine their overlap with the DMN in adults with high and low ADHD ratings.

**Methodology/Principal Findings:**

DC-EEG was recorded using an equidistant 66 channel electrode montage in 25 adult participants with high- and 25 participants with low-ratings of ADHD symptoms during a rest condition and an attention demanding Eriksen task. VLF EEG power was calculated in the VLF band (0.02 to 0.2 Hz) for the rest and task condition and compared for high and low ADHD participants. sLORETA was used to identify brain sources associated with the attention-induced deactivation of VLF EEG power, and to examine these sources in relation to ADHD symptoms. There was significant deactivation of VLF EEG power between the rest and task condition for the whole sample. Using s-LORETA the sources of this deactivation were localised to medial prefrontal regions, posterior cingulate cortex/precuneus and temporal regions. However, deactivation sources were different for high and low ADHD groups: In the low ADHD group attention-induced VLF EEG deactivation was most significant in medial prefrontal regions while for the high ADHD group this deactivation was predominantly localised to the temporal lobes.

**Conclusions/Significance:**

Attention-induced VLF EEG deactivations have intracranial sources that appear to overlap with those of the DMN. Furthermore, these seem to be related to ADHD symptom status, with high ADHD adults failing to significantly deactivate medial prefrontal regions while at the same time showing significant attenuation of VLF EEG power in temporal lobes.

## Introduction

In the last decade, studies of the functional properties of the resting brain have identified a network of connected brain regions that are active during rest, but which deactivate during the onset of goal-directed performance [1], [2], [3], [4]. This network includes medial prefrontal cortex (mPFC), posterior cingulate cortex (PCC)/precuneus and medial, lateral and inferior parietal cortex [5], [6], [7], [8], [9], [10], [11]. Resting activity within this network is thought to represent a neurophysiological baseline and has, therefore, been termed the default-mode network [DMN, 12]. Multiple resting state networks have now been identified [13] and are characterised by distinct patterns of resting connectivity across different regions [14]. For instance, a task-positive network, in contrast to the task-negative aspects of the DMN, is also activated during rest and includes dorsolateral prefrontal cortex (dPFC), inferior parietal cortex (IPC) and supplementary motor area (SMA) [9], [10], regions more commonly reported to be active following the transition to attention-demanding tasks [15]. Activations in the task-positive component of the resting brain appear to be anticorrelated with the task-negative network which, according to one model, supports introspectively focused thought while the task-positive network supports extrospective attentional orienting, possibly associated with environmental threat detection and evaluation [9], [10].

Although the DMN was first identified in the resting brain, activity in this network can persist during the transition to goal directed activity. With the onset of a task requiring focused attention, deactivation of the DMN is typically accompanied by activations in task-specific brain areas required for successful task performance [16]. These deactivations are related to the amount of focused attention a task requires: DMN deactivation is greatest for the most difficult and attention-demanding task conditions [2], [3], and a task or stimulus must be suitably cognitively challenging for DMN deactivation to occur [11]. However, DMN activity may persist even during tasks with high cognitive and attention demands [e.g. 17] under certain conditions and in certain individuals [18]. Where this occurs it may interfere with task-specific neural activity and disrupt performance [17], [19]. For example, Weissman et al. [20] reported that reduced DMN deactivation was associated with lapses in attention (indexed by long reaction times (RTs)). Similarly, Fassbender et al. [21] found that children with attention deficit/hyperactivity disorder (ADHD) had increased reaction time variability (RTV) when they failed to sufficiently deactivate ventral mPFC during a working memory task. Subsequent work has shown that this reduction in task-induced deactivation of DM regions in children with ADHD is ameliorated by the administration of methylphenidate during Stroop task performance [22].

While attention-induced deactivations in the DMN were first identified using positron emission tomography [PET 23], [24], functional magnetic resonance imaging (fMRI) research has shown that functional synchrony across elements of the DMN coheres through brain oscillations at very low frequencies [VLF, <0.1 Hz, [7], [8], [9], [10]]. The functional significance of this VLF characteristic of DMN coherence has been highlighted in a number of models of attention-related disorders [25]. For instance, Sonuga-Barke and Castellanos [18] have proposed that this VLF temporal signature of DMN oscillations has crucial significance for predicting the periodic character of task-related attention and performance. The default-mode interference [DMI, 18] hypothesis predicts that when VLF default-mode activity persists during a task it produces a pattern of periodic lapses in attention and performance also with a VLF temporal structure and created by the low frequency periodic intrusion of the DMN into task-specific activations. To test the functional significance of the VLF signature for attention-related cognitive processes, it is important to explore the temporal correlation between VLF oscillations in DMN activity and attentional performance. Given the temporally constrained nature of the BOLD signal, fMRI studies are of limited value in this endeavour.

Compared to the BOLD signal, DC-EEG allows a direct assessment of oscillatory brain activity with greater temporal resolution and so is a more appropriate vehicle to explore the functional significance of VLF brain activity in the DMN, albeit with more limited spatial resolution. To address these questions we have recently conducted studies examining VLF EEG activity at rest, the patterns of deactivation that occur with the onset of a task, and their relationship with attention. First, VLF EEG has a temporally stable and distinctive spatial distribution across the scalp with maximal power distributed across frontal midline and posterior regions [26], [27]. Second, there is deactivation of VLF EEG power across this scalp network following the transition from rest to task [27], [28]. Third, these deactivations are correlated with attention performance [27]. Fourth, there is a degree of synchrony between VLF EEG and VLF fluctuations in reaction time (RT) data [28]. Finally, as predicted by the DMI hypothesis, adults with high ADHD ratings and adolescents with a clinical diagnosis of ADHD exhibit reduced attenuation of power within these VLF EEG scalp networks following the transition to an attention demanding task [27], [28]. As well as suggesting that the task-related attenuation of VLF EEG oscillations are of some functional and clinical significance, the results suggest similarities between the DMN as identified by fMRI and PET and the VLF EEG scalp network in terms of rest-task deactivation, attention performance [2], [17], [19], [29] and ADHD symptoms [21], [22].

But the fundamental question persists: Is the VLF DC-EEG network identified at the scalp, which deactivates in the transition from rest to attention demanding tasks, generated by the same brain sources involved in the DMN as shown by PET and fMRI. To answer this question, here we extend our previous DC-EEG work by investigating the underlying brain sources that might generate these VLF patterns of EEG activity, and the relationship between these sources and attentional abilities. Therefore, the aim of the current study was to examine patterns of VLF-EEG attention-induced deactivation by comparing VLF EEG at rest and during an attention task. sLORETA was used to localise the sources of these deactivations of VLF EEG power within the brain, and to compare these sources in adults with low and high levels of ADHD. We predicted; (i) that there would be significant attention-induced deactivations in VLF-EEG power and this would be greater for low than high ADHD participants and (ii) that deactivations would be localised to previously identified DM regions including mPFC, PCC/precuneus; (iii) that this would be most marked in the low ADHD group, and that anterior DM sources (e.g. mPFC) would be less significant in the high ADHD group in line with previous fMRI findings [21], [22], [30].

## Results

All participants performed the task with a high level of accuracy (M: 94.5%, SD: 5.1%). Table 1 reports mean (SD) values for error rates, RT and variability measures for each group. Significantly more errors of commission and increased MRTs were recorded for neutral compared with congruent stimuli, and incongruent compared with neutral stimuli. The number of omitted responses was very small and did not differ between stimulus types. For incongruent trials, SD of RT was greater relative to the neutral trial condition, and tended to be greater for neutral compared with congruent trials (*p* = .051). Group differences in performance were limited to errors of omission and were more common in the high than low ADHD group although it must be noted that even for high ADHD participants the mean percentage of omission errors was very small (≤0.5%).

**Table 1:** Performance measures on the Eriksen task.

	Stimulus type						Main effect (Stimulus)				Group effect		M × G			
	Neutral		Congruent		Incongruent		N > C		I> N				N > C		I > N	
	Low ADHD	High ADHD	Low ADHD	High ADHD	Low ADHD	High ADHD	*F*	*p*	*F*	*p*	*F*	*p*	*F*	*p*	*F*	*p*
**Commission %**	1.21 (1.22)	1.71 (1.92)	0.63 (0.93)	1.38 (2.28)	14.54 (10.23)	15.67 (12.12)	4.94	.032	60.71	.001	<1	ns	<1	ns	<1	ns
**Omission %**	0.04 (0.19)	0.33 (0.68)	0.04 (0.19)	0.25 (0.48)	0.13 (0.41)	0.50 (0.78)	<1	ns	1.17	.286	7.11	.011	<1	ns	<1	ns
**Mean RT (ms)**	402.59 (31.29)	413.19 (52.15)	393.10 (30.08)	404.57 (56.34)	490.56 (59.76)	497.37 (51.68)	36.78	.001	237.68	.001	<1	ns	<1	ns	<1	ns
**SD of RT (ms)**	69.64 (15.39)	77.78 (39.49)	66.08 (15.01)	47.25 (38.21)	80.20 (21.36)	90.22 (41.28)	4.08	.051	17.34	.001	<1	ns	<1	ns	<1	ns
**Normalised Var.**	0.17 (0.04)	0.18 (0.07)	0.17 (0.04)	0.18 (.07)	0.16 (0.03)	0.18 (0.07)	1.45	.236	2.24	.143	<1	ns	<1	ns	<1	ns

Notes: N  =  Neutral, C  =  congruent, I  =  incongruent; Commission % and Omission % refers to the percentage of Commission and Omission errors.

Main effect  =  effect of Stimulus type, M × G  =  Stimulus × Group interaction. ^a^ For illustrative purposes, the mean percentage of omission errors for each group, however as this variable was not normally distributed, analyses were performed on the square root transformed data.

**Figure 1:**
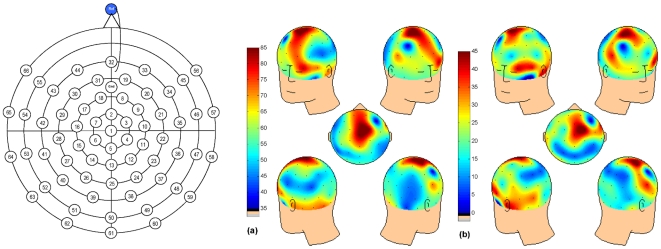
VLF frequency EEG scalp distribution. Spatial distribution of (a) VLF power (0.02–0.2 Hz) at rest and (b) attention-induced deactivation of VLF power for the whole sample. For reference, the electrode montage is shown on the left and topographic maps on right.

Figure 1 (a) shows the spatial distribution of maximal VLF EEG power band at rest in the whole sample. Inspection of this VLF power distribution in the whole sample revealed maximal power in medial frontal regions extending into centroparietal areas. There was no difference between the low and high ADHD group in terms of VLF power at rest or intracerebral sources (*p*>0.100).

**Figure 2:**
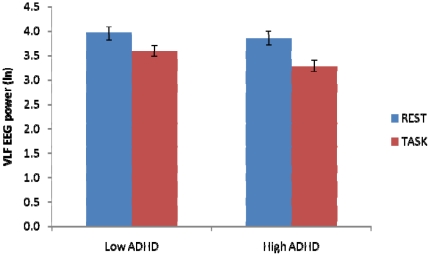
Attention-induced deactivation of very low frequency (0.02–0.2 Hz) EEG in low and high ADHD groups.

**Figure 3:**
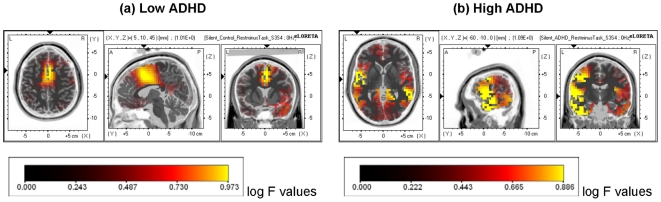
Localisation of attention-induced deactivation of VLF EEG (0.02–0.2 Hz). Localisation of VLF EEG shown for the (a) low ADHD group and (b) high ADHD group. Significant voxels are shown in yellow (*p*<.05).

There was significant attention-induced deactivation of VLF EEG in terms of attenuation between rest and task (*F* (1,38) = 14.13, *p* = .001). There was no significant effect of Group or Group × Condition interaction (all *ps* >0.1; see Figure 2). Figure 1 (b) shows the scalp distributions of the attention-induced deactivation of VLF EEG power in the whole sample. Consistent with the resting state scalp distribution, deactivation of VLF EEG power extended from frontocentral to centroparietal areas, and additionally over bilateral temporal regions. As predicted, s-LORETA identified significant deactivations for the whole sample in medial prefrontal regions including precentral (BA 43), medial (BA 6) and middle frontal gyrus (BA 8), PCC/precuneus (BA 23 and 31) and postcentral gyrus (BA 43). Deactivations were also seen in temporal regions including the superior (BA 22) and middle temporal gyrus (BA 21), transverse temporal gyrus (BA 41 and 42) as well as cingulate gyrus (BA 23, 24 and 32) and parahippocampal gyrus (BA 30; log F = 0.688, extreme *p* = .003). Figure 3 shows the intracranial sources associated with these VLF-EEG deactivations identified using sLORETA for each group. The patterns were markedly different for the low ADHD and high ADHD participants. Deactivations in medial prefrontal regions including the medial frontal gyrus (BA 6 and 32), superior frontal gyrus (BA 8) and cingulate gyrus were found to be significant in the low ADHD but not the high ADHD group (BA 24; log F ratio (1,19)  = 0.973, extreme *p* = .036; see Figure 3(a)). For the high ADHD group significant deactivations were found in temporal lobes including the superior (BA 22) and middle temporal gyrus (BA 21) and fusiform gyrus (BA 37; log F ratio (1,19)  = 0.886, extreme *p* = .002; Figure 3(b)).

## Discussion

The VLF signature of DMN activity has been hypothesised to play a role in periodic attentional lapses. fMRI is poorly positioned to test the validity of this hypothesis. DC-EEG with its superior temporal resolution and direct association with neuronal oscillations may provide a more effective platform to assess the functional significance of these VLF brain fluctuations. Using this method, a temporally stable and robust scalp distribution of VLF EEG oscillations has been identified [26], [27] which, like the DMN, shows deactivation with the onset of attention demanding tasks, which is related to task performance and is reduced in individuals with ADHD [27], [28]. The current study extended this research by isolating the sources of these attention-induced deactivations in VLF neuronal oscillations to regions that overlapped with those identified as the DMN using fMRI and PET imaging, especially the mPFC and the PCC [e.g., 1], [2], [3], [7], [8], [17], [20]. We also found deactivations in other regions including the temporal lobes. While temporal lobe deactivation was not specifically predicted, a relationship between the classic DMN regions and temporal structures has been previously noted. In healthy adults, temporal structures show resting state connectivity with PCC/precuneus [9] and are significantly deactivated by sustained attention [31], an effect enhanced by nicotine [32].

Although the high and low ADHD groups did not differ in terms of the amount of VLF EEG deactivation, there were striking group differences in the localisation of VLF EEG deactivation to specific brain regions. For the low ADHD group, as predicted, the deactivations were localised to medial prefrontal regions, including the cingulate gyrus (BA 23, BA 24, BA 32), medial prefrontal gyrus (BA 6) and superior frontal gyrus (BA 8). The medial prefrontal cortex is a core node within the anterior component of the DMN, and has been especially associated with the self-referential processing role of this network [33]. Although not reaching traditional levels of significance, VLF EEG deactivations also occurred in low ADHD participants in posterior DM regions such as the PCC/precuneus (see Figure 3). Previous research indicates that attenuated task-related deactivation of the BOLD response in PCC and adjacent precuneus predicts response errors in an Eriksen task [17], while reduced connectivity with anterior DM regions has been reported in patients with ADHD [30], [34], suggesting that the PCC may play an important role in attentional control, while other research has also linked PCC/precuneus with working memory function [11]. One explanation for the lack of significant task-related VLF EEG rest-task attenuation in these more posterior regions could be that the task was not challenging enough for these participants. Although Eichele et al. [17] also employed an Eriksen task, these authors utilised a speeded version which adjusted task difficulty on an individual trial and participant basis, ensuring the task was both sufficiently challenging and produced an ample number of errors for their single trial analysis. Manipulating task difficulty and designing paradigms which permit single trial analysis will be important considerations for future research.

Consistent with our predictions and previous fMRI work, deactivations were not significant in medial prefrontal regions of the DMN in the high ADHD group, but rather significant VLF EEG deactivation was identified in middle and superior temporal regions. The lack of deactivation of mPFC is consistent with previous reports of ADHD-related reductions in task-related deactivation of the BOLD response in DM regions during a working memory [21] and Stroop task [22]. In both of these studies, participants with ADHD did not effectively deactivate anterior DM regions including the mPFC [21] and ventral ACC, an effect which was normalised by the administration of methylphenidate [22]. It also overlaps with fMRI work reporting decreased connectivity between this anterior node within the DMN and PCC/precuneus [30], [34]. Indeed, Castellanos et al. [30] note that the anterior node of the DMN was absent in their ADHD sample, and suggests that the strong disconnection between anterior and posterior components may be associated with the attentional and working memory deficits characteristic of this disorder. The deactivations of the temporal lobe are more difficult to place in the context of the current literature. In contrast to our results, two recent studies have highlighted reduced attention-induced deactivation of the temporal lobe during a working memory task in boys with ADHD [35] and during a colour-word Stroop task in young adults with ADHD [36]. Clearly further work examining temporal lobe deactivations needs to be undertaken; the current findings are perhaps more consistent with work showing patients with ADHD exhibit increased VLF resting state BOLD activation and connectivity in sensory cortices including superior temporal gyrus [37] and occipital cortex [38].

The relationship between VLF EEG oscillations and higher EEG frequency bands relating to the DMN deserves future study. Previous simultaneous EEG-fMRI recording studies have reported an association between DMN deactivations and higher frequency bands such as alpha and theta. One such study found that when the difficulty of a working memory task increased, corresponding frontal theta power increases were associated with increased deactivation of BOLD response in medial frontal regions, PCC/precuneus and other DMN regions [39]. Moreover in a different working memory task, Meltzer et al. [40] collected EEG and fMRI data in separate test sessions, and used LORETA to localise frontomedial theta activity to the mPFC and ACC. When theta activity was then correlated with the BOLD signal, the authors reported an association between increased theta and task-induced deactivation of DMN regions, particularly the anterior mPFC [40]. Simultaneous EEG-fMRI currently offers a promising methodological platform with which to clearly establish the relationship between VLF EEG activity and the DMN as defined by fMRI and PET work. To date, previous simultaneous fMRI-EEG work has focused on the relationship between higher EEG frequency bands (e.g. alpha and theta) and default-mode structures [39], [40], [41]. To better understand attentional mechanisms associated with VLF brain activity, it is imperative that future research, both from an EEG and also brain source and dynamics perspective, explores the relationship between these VLF bands and the higher traditional EEG bands. Finally, given the superior temporal resolution of EEG compared to other imaging methods, it is well placed to assess the relationship between oscillatory brain activity and variability in task performance.

This study had a number of limitations which should be addressed in future work: First, future research should examine this relationship under different task conditions and include experimental tasks with frequent sampling (i.e. short ISI) in order to create a near continuous measure of attention. Second, in the current study, the groups did not differ in the degree to which they deactivated VLF EEG power during the attention task: Although participants in the current study were carefully selected on the basis of their ADHD symptom scores using self- and other- report measures, all participants were undergraduate psychology students and therefore high functioning, and without a clinical diagnosis. Future research should replicate the current design and include clinical cases, particularly in light of fMRI research showing atypical attenuation [21], [22] and connectivity within the DMN [30], [34], [37], [42]

In summary, although all participants exhibited equivalent attention and near-equivalent performance on the task, brain generators associated with the attention-induced deactivation of VLF EEG were differentially localised according to ADHD symptom status. Consistent with our predictions, attention-induced deactivation of participants with low ADHD symptom scores could be localised to medial prefrontal regions. In contrast, despite exhibiting equivalent task-related attention, participants with high ADHD did not show mPFC deactivation, but rather VLF EEG deactivation was localised to temporal regions. In conclusion, the findings of this study corroborate fMRI investigations of VLF brain activity reporting atypical deactivation of DM regions in participants with attentional deficits, with a more direct measure of neuronal oscillations and provide a crucial link between VLF EEG and the low frequency fluctuations in the DMN as measured by the BOLD signal.

## Materials and Methods

### Participants

First and second year undergraduate students from the School of Psychology at the University of Southampton were screened for ADHD symptoms with the Adult ADHD rating scale [43]. This scale contains 18 symptom items for ADHD as per the DSM-IV, and includes two related subscales of inattention and hyperactivity/impulsivity. This scale has been shown to possess good construct validity and test-retest reliability (construct validity 35-.85, 4 week test-retest reliability 78-.86; [see 44 for a review]). Participants scoring in the top (>80^th^ percentile; high ADHD group; N = 25) and bottom 20^th^ percentiles (low ADHD group; N = 25) were invited to take part in the study. These scores were validated against the ratings of a relative, partner or close friend of each participant who completed an adapted version of the Adult ADHD rating scale with regards to the participant's behaviour over the preceding 6 months. Items within the friend/relative scale were analogous to those in the self-report scale although adapted so that items such as ‘[I] have difficulty awaiting turn’ would instead read ‘[He/She] has difficulty awaiting turn’. This data was collected for all but 7 participants in the high ADHD group, who failed to return this information to the researcher. The comparison between the self and friend/relative scale was carried out for the subset of participants for which we had complete data. The self and friend/relative report were found to be significantly correlated in terms of total ADHD scores (*r*(33)  = 0.453, *p*<0.01), inattentive symptoms (*r*(33)  = 0.427, *p*<0.05) and hyperactive symptoms (*r*(33)  = 0.446, *p*<0.01). The low and high ADHD group did not differ significantly in age or gender (see Table 2). Participants in the high ADHD group reported significantly more ADHD symptoms, hyperactivity and inattention than the low ADHD group in the self-report questionnaire and were rated as having more ADHD symptoms and inattention in the friend/relative report. Participants were excluded if they had been diagnosed with a neurological disorder, if they had consumed caffeine within the two hours prior to testing, had used any psychotropic substance (illicit or otherwise) in the 24 hours prior to testing, and/or more than once a month in the previous six months. In addition, all participants were required to have normal or corrected-to-normal vision. Five participants were left handed (two in the low ADHD group). The research protocol was approved by the University of Southampton, School of Psychology Ethics Committee.

**Table 2:** Demographics.

	Low ADHD	High ADHD
Total participants	20	20
Mean (SD) age (years)	22.25 (3.19)	20.61 (1.61)
Number (%) males	2 (10)	6 (30)
*Self-report mean (SD)*		
Total score	10.95 (3.14)	28.10 (3.96)**
Total score inattention	5.65 (2.21)	14.05 (2.40)**
Total hyperactivity	5.30 (2.03)	14.05 (3.00)**
*Relative or friend report mean (SD)*		
Total score	9.15 (6.98)	15.62(9.09)*
Total score inattention	4.15 (3.83)	7.92 (4.35)*
Total hyperactivity	5.00 (4.58)	7.69 (6.02)

*p<0.05,

**p<0.001. Where variance between the groups is not equal, the results of the equal variance not assumed test statistic are reported.

### Procedure

All participants were familiarised with the electrophysiology laboratory and EEG recording procedure before informed consent was taken. Participants were asked to complete a short screening questionnaire to assess vision problems, medication and psychotropic substance use, and neurological disorders. They were fitted with recording electrodes and seated in a comfortable chair in the testing room. The halogen light in the testing booth was dimmed for the duration of the experiment. Participants took part in four assessments: two five minute eyes-open resting conditions and two 10 minute Eriksen flanker tasks. Each resting condition and Eriksen task was completed once in silence and once while the participant listened to 80 dB of white noise through headphones. Participants completed the tasks either second or third, and the resting conditions first or last, in a counterbalanced order. Here we report data recorded during the silent rest and task conditions only.

Prior to each resting condition, participants were instructed to sit quietly and relax and focus their gaze on a fixation cross in the centre of a computer screen, positioned 60 cm in front of the participant. The Eriksen tasks consisted of two five minute blocks, with a short break in between. In this task participants were presented with a combination of five left- and/or right-pointing arrows and equals signs presented centrally on the computer screen for 200 ms (variable ISI, M = 3050 ms). The central arrow in the array is the target, while the flanking distractors are either four left- or right-pointing arrows, or four equal signs. Congruent trials were those in which the target arrow is flanked by distractor arrows pointing in the same direction as the target (e.g. <<<<< or >>>>>). Incongruent trials were such that the target arrow is flanked by distractor arrows that are pointing in the opposite direction to the target (e.g. <<><< or >><>>). On neutral trials the target arrow was flanked by equals signs (e.g.  =  = < =  =  or  =  = > =  = ), which were chosen for their identical spatial size, and because they do not contain any directional information. Participants were required to respond to the centrally presented arrow, such that a left-pointing target was associated with a left-handed button press and a right-pointing target was associated with a right-handed button press.

### Electrophysiological acquisition and processing

An electrode cap (Easycap, Herrsching, Germany) containing 66 equidistantly spaced silver/silver chloride (Ag/AgCl) electrodes was fitted to each participant and EEG data was recorded using Neuroscan Synamps^2^ 70 channel EEG system, DC-coupled recording equipment. The data were sampled at 250 Hz with a low pass filter at 70 Hz and referenced to an electrode on the nose. A ground electrode was fitted midway between the electrode at the vertex and frontal sites. Vertical electro-oculogram (vEOG) was recorded from four electrodes: two bipolar electrodes were placed directly beneath the left and right eyes and affixed with tape, while the two electrodes placed above the right and left eye were included within the electrode cap. Impedances for vEOG, reference and cap electrodes were kept below 5 kΩ. EEG data were analysed using MATLAB (version R2008b) and re-referenced to an average reference. Using the ‘detrend’ command in MATLAB, the linear trend caused by drift was removed and the first 55 of 66 electrodes were selected for further analysis (see Figure 1). Independent Component Analysis (ICA) was used to remove all artifacts (including ocular movements) from the data. This analysis was performed separately for the rest and task condition. The EEG signal was then reconstructed by back-projection of all artifact-free components. Ten participants (five in each group) were excluded because of poor data including excessive movement artifacts, these artifacts continued to obscure the EEG after ICA had been performed, so final comparisons were conducted on two groups of twenty (N = 40).

### Data analysis

#### We first examined performance data

Errors of commission and omission, as well as mean reaction time (MRT), standard deviation of reaction time (SD of RT) and normalised variance (SD of RT/MRT) were recorded for each participant. A 3×2 ANOVA was used to compare error and reaction time data with Stimulus (Neutral, Congruent, Incongruent) as the within-subjects factor and Group (low ADHD, high ADHD) as the between subjects factor. Simple planned contrasts compared neutral with congruent and incongruent trials.

#### We then examined VLF EEG during rest

EEG data from each participant for 55 electrodes were subjected to Fast Fourier Transformation (FFT) for the five minute resting condition and the first five minute block of the Eriksen task. Building on our previous research [26], [27], the current analysis focused on VLF EEG band (0.02–0.2 Hz). To this end, one minute Hamming windows that overlapped by 20 seconds were used and power (as indexed by the area under the curve) was calculated in the combined slow 3 (0.06 to 0.2 Hz) and slow 4 (0.02 to 0.06 Hz) bands [45] for each condition. The natural log transform of the power in each frequency band was calculated as power is not normally distributed [46]. Using this data we plotted the spatial distribution of VLF EEG power across the scalp at rest (see Figure 1). Using an independent samples t-test we compared the groups in terms of VLF EEG power at rest.

#### Finally we explored deactivation at the scalp level and also in terms of the localisation of specific brain sources

We examined the attention-induced deactivations of VLF EEG power by comparing power in the rest and task condition for the whole sample and for the high and low ADHD groups. The scalp distribution of the deactivation of VLF EEG power is also shown in Figure 1. We tested for differences using a 2×2 repeated measures ANOVA with Condition (rest and task) as the within-subject factor and Group (low ADHD and high ADHD) as the between subjects factor. The analysis of intracranial sources was based on s-LORETA analysis [47], [48] using standardised current density and the EEG data from 55 electrodes with an equidistant distribution. The sLORETA method is a properly standardised, discrete and 3D distributed, linear and minimum norm solution with no localisation bias even in the presence of biological or measurement noise [47], [49]. Because of the computational constraints of the sLORETA method, the ICA-cleaned EEG data was downsampled from 250 Hz to 25 Hz in MATLAB using the ‘decimate’ command. The five minute artifact free epochs of task and rest data were then exported in ASCII format from MATLAB for sLORETA analysis. Using sLORETA, the EEG cross-spectra for the VLF band (.02 to 2 Hz) were calculated for the rest and task conditions. Next, using the cross-spectra we calculated the cortical generators of the scalp-recorded EEG activity. We used sLORETA to calculate the log F ratio differences between each cortical voxel and compare the generators associated with the attenuation, or deactivation of VLF power during goal-directed attention and task performance first in the entire sample, and secondly in the low and high ADHD groups separately.
